# Editorial: Predicting Individual Responses to Exercise Interventions, Volume II

**DOI:** 10.3389/fphys.2022.850919

**Published:** 2022-02-18

**Authors:** Brendon Gurd, Giuseppe D'Antona, Vassilis Mougios

**Affiliations:** ^1^School of Kinesiology and Health Studies, Queen's University, Kingston, ON, Canada; ^2^Department of Public Health, Experimental and Forensic Medicine, CRIAMS-Sport Medicine Centre Voghera, University of Pavia, Pavia, Italy; ^3^Laboratory of Evaluation of Human Biological Performance, School of Physical Education and Sport Science, Aristotle University of Thessaloniki, Thessaloniki, Greece

**Keywords:** exercise training, interindividual variability, genomics, proteomics, metabolomics

After a successful first volume on the Research Topic of predicting individual responses to exercise interventions in 2020, Frontiers in Physiology has launched and completed a second volume. As with the original volume, the aim is to explore the issue of interindividual differences in the biological responses to exercise training, aiming at the direction of discovering predictors that will allow more accurate prescription of exercise regimens and thus maximize the health benefits of exercise. A total of three original papers and two reviews have been included in the present volume.

Christou et al. explored the anthropometric, cardiorespiratory, and hematological determinants of marathon performance. By assessing male and female marathon runners during the training period for a race, they identified low body fat percentage and right ventricular enlargement as the main physiological determinants of better marathon performance. Upregulation of maximum minute ventilation during exercise and estimated hemoglobin mass were proposed as weaker predictors of marathon performance.

Ojeda-Aravena et al. investigated the interindividual variability (in terms of responders and non-responders) in the effects of two 4-week training programs (high-intensity interval training with either specific techniques or repeated sprints) on sport-related fitness of taekwondo athletes of both genders. They found a higher proportion of responders to the specific-technique program, compared to the repeated-sprint program, with regard to improvement in some characteristics of lower limb performance (squat jump, countermovement jump, and total kicks).

Zapata-Bustos et al. studied differences in the expression of transcription factors in response to acute exercise between sedentary lean and obese men and women, the obese group displaying insulin resistance. By analyzing muscle biopsies obtained pre- and postexercise, they identified several transcription and growth factors with different responses to exercise between groups. Specifically, an increase (EGR3 and CTGF) and decrease (RELA and ATF2) in the mRNA of transcription and growth factors was found after exercise in the lean but not in the obese participants. These findings highlight obesity and insulin resistance as modulators of the biological responses to exercise.

Herold et al. focused on interindividual response variability in the field of exercise-cognition. In their review, they discuss the causes and consequences of interindividual variability, critically examine studies that have investigated interindividual variability of neurocognitive outcomes in response to acute exercise, and provide recommendations for future studies. Basically, they call for more rigorous study designs that define and report the exact exercise prescription in terms of external and internal load, as well as controlling for non-modifiable (such as sex and age) and modifiable factors (such as exercise parameters, diet, and sleep). Such designs will allow researchers to draw robust conclusions and, based on them, provide accurate practical recommendations.

Finally, Appel et al. performed a systematic review of 20 studies that have examined the effects of genetic variation on endurance performance, muscle strength and injury susceptibility in sports. They report that polymorphisms of IGF-1R and PPARGC1A may be correlated with endurance performance, polymorphisms of ACTN3, IGF-1R, FTO, MCT1, and ACVR1B may be associated with muscle strength, while polymorphisms of MMP and COL5A1 may be related to susceptibility of competitive athletes to injuries. The authors propose that individualized training programs for injury prevention and optimization of athletic performance could be created on the basis of the identified gene variants.

In conclusion, the articles included in this Research Topic highlight, once more, how complex the factors that determine individual responses to exercise interventions are. Future research in this area could address the following issues:

- The need to reduce the number of variables affecting the individual responses to exercise. In particular, it is necessary to reduce the impact of factors such as nutrition, the basal level of physical activity, sleep patterns, and (particularly in female individuals) hormonal fluctuations. From this point of view, a fundamental role can be entrusted to the wise use of artificial intelligence without a priori hypotheses.
- The need to identify additional biomarkers of the individualized responses to exercise by crossing new frontiers through omics technologies.
- The urgent need to investigate these issues within the framework of individual responses to exercise in disease states to fully exploit the therapeutic potential of exercise.

We feel that the time is ripe to go deeper inside the mechanisms behind the different responses in diverse individuals instead of merely describing them. We hope that perusing this collection will benefit and stimulate interested readers and researchers. Feedback is most welcome.

## Author Contributions

All authors listed have made a substantial, direct, and intellectual contribution to the work and approved it for publication.

## Conflict of Interest

The authors declare that the research was conducted in the absence of any commercial or financial relationships that could be construed as a potential conflict of interest.

## Publisher's Note

All claims expressed in this article are solely those of the authors and do not necessarily represent those of their affiliated organizations, or those of the publisher, the editors and the reviewers. Any product that may be evaluated in this article, or claim that may be made by its manufacturer, is not guaranteed or endorsed by the publisher.

